# Oligodendrocytes in Development, Myelin Generation and Beyond

**DOI:** 10.3390/cells8111424

**Published:** 2019-11-12

**Authors:** Sarah Kuhn, Laura Gritti, Daniel Crooks, Yvonne Dombrowski

**Affiliations:** Wellcome-Wolfson Institute for Experimental Medicine, Queen’s University Belfast, Belfast BT9 7BL, UK; skuhn01@qub.ac.uk (S.K.); l.gritti@qub.ac.uk (L.G.); dcrooks03@qub.ac.uk (D.C.)

**Keywords:** oligodendrocytes, OPC, oligodendrocyte progenitor cells, myelin, myelination, remyelination, multiple sclerosis

## Abstract

Oligodendrocytes are the myelinating cells of the central nervous system (CNS) that are generated from oligodendrocyte progenitor cells (OPC). OPC are distributed throughout the CNS and represent a pool of migratory and proliferative adult progenitor cells that can differentiate into oligodendrocytes. The central function of oligodendrocytes is to generate myelin, which is an extended membrane from the cell that wraps tightly around axons. Due to this energy consuming process and the associated high metabolic turnover oligodendrocytes are vulnerable to cytotoxic and excitotoxic factors. Oligodendrocyte pathology is therefore evident in a range of disorders including multiple sclerosis, schizophrenia and Alzheimer’s disease. Deceased oligodendrocytes can be replenished from the adult OPC pool and lost myelin can be regenerated during remyelination, which can prevent axonal degeneration and can restore function. Cell population studies have recently identified novel immunomodulatory functions of oligodendrocytes, the implications of which, e.g., for diseases with primary oligodendrocyte pathology, are not yet clear. Here, we review the journey of oligodendrocytes from the embryonic stage to their role in homeostasis and their fate in disease. We will also discuss the most common models used to study oligodendrocytes and describe newly discovered functions of oligodendrocytes.

## 1. Introduction

Oligodendrocytes are the myelinating cells of the central nervous system (CNS). They are generated from oligodendrocyte progenitor cells following tightly orchestrated processes of migration, proliferation and differentiation [1]. Oligodendrocytes are fundamental to myelin formation in the developing CNS and critical for myelin regeneration following injury including in the most common demyelinating disease multiple sclerosis (MS) [2,3]. In this review, we discuss the journey of oligodendrocytes in development from the embryonic stage to oligodendrocyte function in health and their fate in disease. We will also review the most common models used to study oligodendrocyte behaviour and function and discuss recent advances in our knowledge of oligodendrocytes.

## 2. Oligodendrocyte Progenitor Cells (OPC) and Oligodendrocytes in Development

### 2.1. OPC and Oligodendrocytes during Embryonic Development

Oligodendrocytes are one of the major glial cell types in the CNS besides microglia and astroglia. Oligodendrocytes were first described by Virchow [4], Deiters [5] and Golgi [6] in the 19th century. While neurons had been well characterised at that time, an abundant unknown cell type called “neuroglia” became the focus of attention. Analysing the fine structure of the brain led Virchow to introduce the term “Nervenkitt” (German: nerve glue) as cells appeared in between nerves [4,7]. Distinct from neurons, these cells could not grow axons [5] and were able to proliferate even postnatally. Initially, they were not thought to have a role other than being connective tissue. Pio del Rio-Hortega eventually differentiated neuroglia into microglia and four types of oligodendroglia by using more advanced staining techniques, including silver carbonate [8,9].

Amongst the glial populations, bipolar cells were found to be highly proliferative and migratory, whereas filamentous myelin-producing glia were mainly found in white matter suggesting a bipolar precursor cell type, later termed oligodendrocyte progenitor cell (OPC), and a distinct, differentiated cell type, the oligodendrocyte [10].

The origin of neurons was well characterised within the CNS yet less was known about the origin of oligodendrocytes and microglia [11,12]. Microglia originate from the yolk sack [13], while oligodendrocytes develop from multiple origins in the brain and anterior spinal cord with radial glial cells, the ventral ventricular zone and dorsal spinal cord as sources [14,15,16,17,18,19]. Neural progenitor cells (NPC) arising in the neural tube during embryonic development are the common precursors for oligodendrocytes, astrocytes and neurons [20,21].

Murine OPC in brain and spinal cord are characterised by the expression of DM-20 mRNA [16,22]. Using this marker, three temporal waves of OPC development were identified starting in the ventral ventricular zone after neural tube closure from E9.5 [16]. Rat OPC first appeared at around E14 [17] and human OPC at E45, reflecting the gestational week 6.5 [23]. Across species, several waves of OPC generation were identified. The first wave of OPC generation in the forebrain is followed by a smaller second wave from the dorsal ventricular zone and by a third postnatal wave originating in the cortex [24]. In the spinal cord, ventrally-derived OPC are followed by a wave of dorsally-derived OPC [25,26], which make up to 20% of total OPC [19,25].

These waves subsequently lead to an overproduction of progenitor cells, which compete for space and survival factors provided by astrocytes and axons [24,27,28,29]. It was shown that most OPC from the first cortical wave die [24,27]. However, Orduz et al. recently demonstrated that a subpopulation of first-wave cortical OPC not only survive but have non-redundant functions [30].

In the adult normal CNS, the majority of proliferating cells are of oligodendroglial lineage origin, which by dividing self-renew and generate mature oligodendrocytes, whereas little evidence exists that neurons are able to proliferate in adulthood [31,32,33,34].

During vertebrate development, one of the major and best-established morphogens is the Sonic hedgehog (Shh) protein, required for most OPC to originate during early embryogenesis [26,35]. Shh is produced by the notochord, which is essential for the CNS dorsal–ventral axis development [36,37]. OPC as well as motor neurons originate from similar areas in the ventral neural tube during development and need equal amounts of Shh in order to arise, which suggests an interlinked generation of both cell types [38,39,40]. However, in vitro experiments with Shh deficient cells indicated that Shh is not an absolute requirement for OPC origination [41], while mice lacking a notochord did not develop any OPC [38]. Cai et al. corroborated these findings in vivo demonstrating *Nkx6-* and *Shh*-independent generation of an OPC subpopulation, which suggests the involvement of other molecular pathways [26]. Indeed, *Shh* is important for the timing of OPC generation as a recent study has shown [42].

Tightly regulated epigenetic mechanisms, such as DNA methylation and histone modification, have recently been discovered in the regulation of OPC differentiation that are distinct in the different developmental stages and in myelin regeneration (reviewed in detail in [43] ).

More recently, activated neurons were shown to play a role in the origination and proliferation of OPC, and oligodendrocytes to myelinate [44,45,46,47].

### 2.2. Distribution of OPC and Oligodendrocytes within the CNS

Only 5%–8% of total glial cells are OPC [48], which are evenly distributed in white (WM) and grey matter (GM), with OPC being slightly less abundant in GM [48]. The location gives rise to behavioural differences between WM and GM OPC; while WM NG2^+^ OPC in organotypic brain slices had a greater proliferative response to PDGF-A, GM OPC were less responsive to PDGF-A and morphologically and genetically less mature than WM OPC [49,50]. In vivo, more WM OPC differentiate into myelinating oligodendrocytes than GM OPC, many of which remain NG2^+^ progenitors as shown by Dimou et al. [51,52], suggesting a potential backup pool of OPC during adulthood. In the adult CNS, oligodendrocyte generation from OPC is slowed down and WM OPC generate about 20% of total differentiated and myelinating oligodendrocytes in the murine corpus callosum vs. 5% in the cortex [53]. However, 20% of cortical GM oligodendroglial lineage cells are differentiated CNP^+^ NG2^-^ oligodendrocytes yet these cells do not myelinate [53]. Recently, Hughes et al. demonstrated that cortical NG2^+^ cells are highly dynamic, balancing their population by proliferation, differentiation and self-repulsion to maintain homeostasis [54].

In order for axonal myelination to occur, migration of OPC from their site of origin into the developing WM tracts of the CNS is required [55]. To overcome this spatial distance, OPC migrate in a jumping or crawling mode along blood vessels within the CNS, which is dependent on WNT signalling [56,57]. Their subsequent excessive proliferation, especially in the WM, leads to an abundant pool of progenitors throughout the brain and spinal cord [58].

### 2.3. Developmental Markers of OPC and Oligodendrocytes

**Figure 1:**
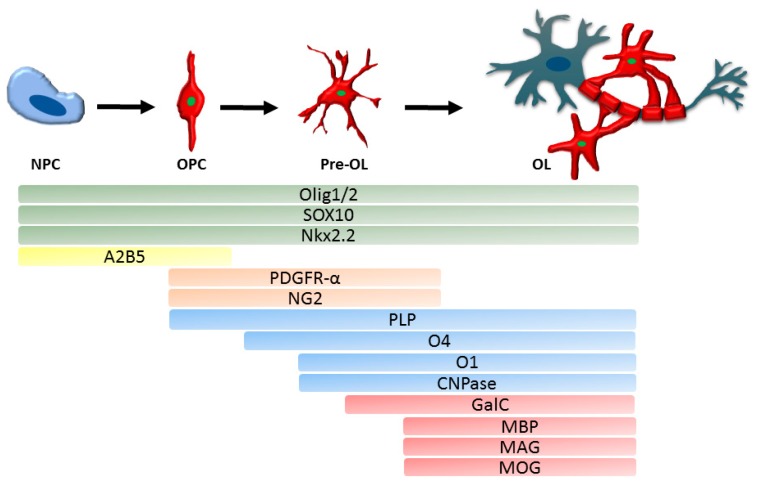
Schematic depiction of oligodendroglial lineage markers specific for different developmental stages from neuronal progenitor cells (NPC) to myelinating oligodendrocyte (OL). A2B5 recognises progenitor cells, NPC and oligodendrocyte progenitor cells (OPC), while oligodendroglial cell lineage markers Olig1 and 2 as well as Sox10 and Nkx2.2 are expressed in all cells of the lineage, OPC and pre-oligodendrocytes (pre-OL) are characterised by PDGFR-α and NG2 expression. PLP, O4, O1 and CNPase are expressed during transition from progenitor to differentiated oligodendrocytes, while differentiated, axon-myelinating oligodendrocytes are characterised by myelin protein expression (MBP, MAG, MOG, GalC). NPC: neuronal progenitor cell; OPC: oligodendrocyte progenitor cell; OL: oligodendrocyte; PDGFR-α: platelet-derived growth factor receptor A; NG2: neuron-glial antigen 2; PLP: proteolipid protein; CNPase: 2’,3’-Cyclic-nucleotide 3’-phosphodiesterase; MBP: myelin basic protein; MAG: myelin associated glycoprotein; MOG: myelin-oligodendrocyte glycoprotein; GalC: galactocerebroside.

New-born OPC are characterised by the expression of DM-20 mRNA, an isoform of protein proteolipid protein (PLP), the most abundant myelin protein [16]. There are numerous additional markers that determine the oligodendroglial cell lineage and reflect their developmental stage, the most prominent are summarised in Figure 1. Once committed to the oligodendroglial lineage, cell surface antigens can be recognized by specific antibodies such as A2B5 [59]. In vitro, A2B5 positive cells can differentiate into both oligodendrocytes and astrocytes, which were therefore termed oligodendrocyte-type-2 astrocyte (O-2A) progenitor cells [60]. O-2A progenitor cells constitutively differentiate into oligodendrocytes unless specific environmental cues redirect differentiation into astrocytes [61].

The best characterised marker for OPC is PDGFR-α, the receptor for PDGF-A, the most potent OPC mitogen and survival factor, which is produced by both astrocytes and neurons [15,62,63,64]. Consequently, overexpression of this growth factor, e.g., during development, leads to increase in OPC numbers [64].

Pre-oligodendrocytes engage with a target axon, thereby losing their bipolarity, and start to build filamentous myelin outgrowths. At this differentiation stage, pre-oligodendrocytes are characterised by the expression of three main myelin associated markers, 2′, 3′-cyclic-nucleotide 3′-phosphodiesterase (CNPase) and the cell surface markers O4 and O1 [65,66]. CNPase has two different isoforms which are differentially expressed: oligodendrocyte precursors only seem to express the larger isoform, whereas myelinating oligodendrocytes were shown to express both isoforms [67]. O4 is already expressed in late progenitors, whereas O1 is typical for pre-myelinating oligodendrocytes [68] (Figure 1).

Mature, differentiated oligodendrocytes are characterised by the production of myelin and myelin proteins, and in combination with a cell lineage specific marker such as Olig2 can be used to identify this maturation stage (Figure 1). The myelin proteins include myelin basic protein (MBP) [69,70], which is expressed on the cytoplasmic surface of the plasma membrane [71], the transmembrane protein PLP [16,72], myelin associated glycoprotein (MAG) [73] as well as the membrane marker galactocerebroside (GalC) [74] and surface marker myelin-oligodendrocyte glycoprotein (MOG) [69]. MBP and MAG first appear between postnatal day 5 and 7 in murine CNS-derived oligodendrocytes, whereas MOG emerges one to two days later [75]. Interestingly, PLP was recently described to be expressed in murine Olig2^+^PDGFR-α^+^ cells, which makes it an early marker for OPC with a role in process extension [76].

Two genetically related, but functionally different, transcription factors Olig1 and Olig2 are present throughout the oligodendroglial lineage [77,78]. Olig2 is essential for NPC to develop into OPC [79] and Olig2 gain of function in OPC can promote remyelination in mice [80]. The role of Olig1 is less well known. Olig1 has a non-redundant role in OPC differentiation and myelination in murine brain development; however, spinal cord OPC are less dependent on Olig1 for differentiation and myelination [81,82]. In repair, however, Olig1 deficient mice showed delayed oligodendrocyte differentiation and impaired remyelination of demyelinated CNS lesions [83]. Another transcription factor involved in oligodendrocyte development that characterises the entire oligodendroglial lineage is SOX10 [84]. SOX10 is essential for NPC derived oligodendroglial lineage specification during early development and for OPC differentiation [85,86,87], while transcription factor Nkx2.2 promotes and regulates timing of oligodendrocyte differentiation [88,89]. A marker for early oligodendroglial lineage cells is the proteoglycan NG2, however, NG2^+^ cells are able to differentiate into oligodendrocytes but also into astrocytes [90]. Antibodies against the markers described here are commercially available.

### 2.4. Other Myelin-Producing Cells

Schwann cells are the oligodendrocyte counterpart in the peripheral nervous system (PNS), derived from the neural crest [91]. Oligodendrocytes and Schwann cells share main functions in providing support and insulation for axons. However, Schwann cells are only able to myelinate single axons rather than multiple axons contrary to oligodendrocytes (reviewed in [92,93]). This feature is dependent on an E3 ligase component, deficiency of which leads to increased myelination potential in murine Schwann cells in vivo [94].

Schwann cells as well as oligodendrocytes are region specific for the CNS and the peripheral nervous system and a glial barrier at the motor exit points was found to prevent oligodendrocytes exiting from the CNS. The cells forming this barrier are motor exit point (MEP) glia, which were recently described as the third cell type capable of producing myelin [95,96]. MEP glia share communalities with both oligodendrocytes and Schwann cells; like OPC, they are derived from the ventral neural tube and they express Olig2 as do oligodendroglial lineage cells. MEP glia are also characterised by the expression of SOX10 and WIF1. This, combined with Olig2 and Foxd3 expression, identifies this population [96]. Similar to Schwann cells, MEP glia express Foxd3 and they are able to myelinate just one axon, although the molecular mechanism underlying the process is different. MEP glia lack *krox20*, a key initiator of myelination for Schwann cells, and are also not affected by *gpr126* deficiency as opposed to Schwann cells. Moreover, MEP glia selectively myelinate spinal motor root axons [95,96,97] and were described to have a role in preventing the migration of OPC into the periphery by blocking off OPC in the CNS via direct contact [96].

## 3. Oligodendrocyte Function in Health

### 3.1. Myelination of Axons

**Figure 2:**
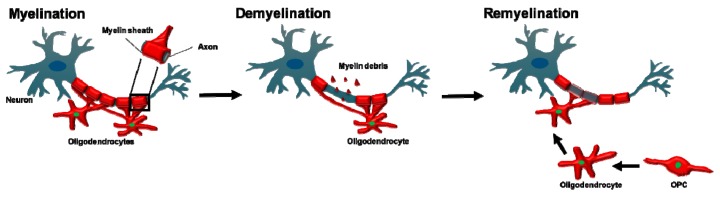
Oligodendrocytes in myelination, demyelination and remyelination. Oligodendrocytes myelinate large diameter axons in the CNS and provide trophic support for the underlying axon. Oligodendrocytes are highly vulnerable and insults such as trauma, immune-mediated attacks or ischaemia can lead to oligodendrocyte death and demyelination. Newly differentiated oligodendrocytes derived from an adult OPC pool can replace deceased oligodendrocytes, which can reinstate the myelin sheath around demyelinated axons (remyelination). Regenerated myelin is thinner than the original myelin sheath.

The myelin sheath is an extension of the oligodendrocyte and Schwann cell plasma membrane that wraps around nerve axons in a concentric fashion [98] as shown in Figure 2. In 1717, Leeuwenhoek described a ‘nervule’ with ‘fatty parts’ around it, likely being the first ever description of myelin [99].

Since then, technical advances in histology and optical techniques have allowed for both the structure and function of the myelin sheath to be explored in detail [3,98,100]. For example, high-resolution time-lapse live imaging, electron microscopy and magnetic resonance imaging of the dynamic processes of myelination and remyelination in vivo have been hugely beneficial to the advancement of our understanding of oligodendrocyte biology in health and disease [3,98,101].

Myelination is a complex and tightly regulated process (reviewed in [102]). In vivo time-lapse imaging in transgenic zebrafish revealed that oligodendrocytes continually extend and retract their processes towards axons before settling into their final positions [103]. The oligodendrocyte processes also sense neighbouring cells to ensure uniform spacing of myelin segments with evenly spaced nodes [104]. Once a mature oligodendrocyte connects with an axon, the oligodendrocyte plasma membrane architecture changes rapidly. Numerous hypotheses have been suggested to explain how the oligodendrocyte extends its plasma membrane to wrap axons and form a compact myelin sheath [99]. Two of the most prominent hypotheses suggest that myelin either extends an inner tongue repeatedly around the axon [105] or alternatively that ‘croissant-like’ layers of myelin sheath are formed on top of pre-existing ones [106]. Sophisticated studies that combined high resolution in vivo imaging and 3D reconstructions of optic nerve fibres fixed with high pressure freezing facilitated improved visualisation of the dynamic process of myelination [107]. These studies revealed that myelination occurs via plasma membrane extension laterally down the axon to form the paranodal loops [107], a discovery consistent with the original mechanism proposed by Geren and Schmitt [105].

Myelination is a highly regulated process that is governed by several molecular cues. Only axons with a large diameter are myelinated [108,109,110], and myelination itself increases the axonal diameter not just simply by the extra myelin sheaths around the axon, but also due to localised neurofilament accumulation and phosphorylation [111,112].

Myelin sheath formation is regulated by several factors such as neuregulin 1 in Schwann cells [113] and Ca^2+^ activity in oligodendrocytes [101], as well as by neuronal activity, which can identify axons for myelination [46]. Intriguingly, neuronal activity regulates also OPC proliferation, differentiation and survival [44,45,46,47].

### 3.2. Functions of the Myelin Sheath

Functionally, the myelin sheath facilitates rapid transmission of axon potentials and provides metabolic support to the axons it ensheaths [3,100]. Sodium channels are located at the intermittent interruptions (nodes of Ranvier), where short portions of the axon are left unwrapped [100,114]. Myelin is an electrical insulator and when the membrane at the node is excited, the axon potential ‘jumps’ from one node of Ranvier to the next, due to the low capacitance of the sheath, in a process called saltatory conduction. Little energy is required to depolarise the remaining membrane between the nodes given the low capacitance of the sheath, which results in transmission of the action potential [100,115]. This form of transmission is much faster than in non-myelinated axons [3].

Oligodendrocytes and the myelin sheath metabolically support axons. Oligodendrocytes can generate lactate, which can then be transferred to axons to generate metabolic energy in the form of ATP [116]. In the brain, lactate is shuttled through the most abundant lactate transporter MCT1, and both MCT2 and MCT4 [117]. MCT1 expression has been detected in endothelial cells, however oligodendrocytes and the myelin sheath also harness MCT1 to transport lactate to axons [117,118]. Lee et al. depleted *MCT1* gene expression in spinal cord cultures, which led to extensive neuronal death; an effect that could be rescued with exogenous lactate supplementation [117]. Indeed, a number of glycolytic and Krebs cycle enzymes such as succinate dehydrogenase and fumarase are expressed in the myelin sheath, which contribute to glucose catabolism and ATP production [119].

The central function of oligodendrocytes in the CNS is the generation of myelin during development, adaptive myelination in adulthood and remyelination after damage, while OPC predominantly serve as a backup pool to generate new oligodendrocytes. However, CNS remyelination can also be mediated by infiltrating Schwann cells, which was described for multiple conditions including MS and traumatic brain injury [2]. Post-developmental OPC can also differentiate into other neural lineage cells such as astrocytes and Schwann cells [120]. OPC-derived Schwann cells reside in CNS regions deficient in astrocytes, suggesting that severely demyelinated lesions that are partially necrotic may inhibit astrogliosis, and that a lack of astrocytic scarring may encourage Schwann cell infiltration [120,121]. The factors governing transition from OPC to Schwann cells remain elusive [2], but these data suggest that an astrocyte-derived factor is necessary for OPC differentiation into oligodendrocytes [120].

### 3.3. Non-Myelinating Functions of Oligodendrocytes and OPC

Emerging evidence suggests OPC have an immunomodulatory capacity. OPC express cytokine receptors [122,123,124] and assess their microenvironment through filopodia extension [103]. In response to inflammatory cues, OPC can migrate to sites of injury, in a manner similar to that of microglia [54,125]. Upon exposure to IFNγ, OPC cross-present antigen to cytotoxic CD8^+^ T cells in vitro and in vivo, leading to their cytotoxic death [126,127]. This newly discovered pro-inflammatory OPC phenotype promotes tissue damage and blocks remyelination. This suggests that suppression of OPC-mediated inflammation may ameliorate cell death in favour of promoting OPC differentiation into myelin-producing oligodendrocytes [127].

## 4. Models to Study Oligodendrocytes and OPC

### 4.1. Rodent and Human In Vitro Models

Several protocols have been developed to isolate and culture oligodendrocytes from rodent brains at different stages from embryonic into adulthood [128,129,130]. The isolation of OPC and oligodendrocytes from murine CNS tissue is based on the expression of markers typical of this maturation stage, for example PDGFR-α or A2B5 for OPC or O4 or GalC for oligodendrocytes [129,130]. These primary cultures are suitable for studying the proliferation, survival and differentiation of OPC as well as the effect of molecules of interest on OPC and oligodendrocyte biology. Moreover, different protocols have been developed to study myelination by co-culturing oligodendrocytes with neurons or synthetic nanofibers [110,131]. Primary OPC cultures derived from e.g., rodents, are particularly interesting for studying the proliferation and differentiation of OPC and are suitable for high throughput screening of pharmacological compounds that may interfere with these processes [132].

However, primary OPC cultures are restricted by the availability of animals and related ethical issues. To overcome these limitations, several laboratories developed spontaneously immortalised cell lines derived from O-2A rat precursors, such as CG-4 or OLN-93 immortalised OPC [133,134], or primary murine OPC immortalised by viral infection, such as Oli-neu [135]. These cell lines are karyotypically normal, express the marker typical of their developmental stages and they can be differentiated into mature oligodendrocytes by withdrawing mitogenic factors, such as in CG-4, or by adding factors, such as dibutyryl cAMP for Oli-neu and OLN-93 [133,134,135]. Immortalised cell lines have undeniable advantages, such as the consistency, the robustness and the low costs related to the model, but because of the unpredictable nature of mutations that lead to the immortalisation, any assumption and translation to any physiological function need to be corroborated by an additional model.

Although the biology of oligodendroglial cells is conserved between rodents and humans, there are some differences between species that should be considered for any translation from murine models to the human physiology (reviewed in [136,137]). The peculiar structure and position of the brain and spinal cord reduces the possibilities of obtaining cells directly from human CNS tissue. One strategy that can be adopted is to isolate OPC from surgery biopsies, e.g., from healthy resection margin of brain tumours (excluding the tumour itself), resected tissue from epileptic or traumatic brain tissue [138]. Another strategy involves the use of post mortem brains or spinal cords; as cells can be isolated up to 12 h after death, from any area of the CNS [139]. Adult human oligodendrocytes can be cultured for 2–3 weeks or directly used for further analysis (for example for mRNA or protein analysis). Although these approaches are precious to unravel the differences between rodent and human biology, the availability of these tissues and the possibility to isolate only oligodendrocytes are a limiting factor.

In recent years, the establishment of induced pluripotent stem cells (iPSC) overcame the restricted availability of human CNS biopsies [140]. iPSC are reprogrammed from somatic cells (e.g., fibroblasts, peripheral blood mononuclear cells) that can be obtained in a non-invasive way. iPSC can subsequently be differentiated to any cell type of interest by inducing their differentiation via specific growth factors (e.g., T3, NT3, IGF, PDGF-A for inducing OPC) or inducing the expression of transcription factors necessary for the transition (e.g., SOX10, Olig2 and NKX6.2 for OPC/oligodendrocytes) [141,142,143]. Recently, protocols with increasing efficiency and reduced time to obtain oligodendrocytes from human iPSC (iOL) were developed; from 170 days in vitro to 20 days to obtain a culture with mature oligodendrocytes (around 70% efficiency of which 20% expressing MBP) ([141,142,143] and reviewed in [137]). The possibility of having OPC and oligodendrocytes derived from healthy donors and patients with neurological diseases as a model of study made iPSC popular in the last decade as proven by the variety of protocols developed. Moreover, the possibility of following the differentiation of OPC into iOL is a valuable tool to study this process in a human system. iOL can also represent a precious model to screen drugs promoting differentiation and/or myelin production directly on human cells, possibly directly derived from patients, as exemplified by Ehrlich et al. [142]. Unfortunately, the elevated costs and the time necessary to obtain iOL reduce the accessibility to this technique on a routine basis in the majority of laboratories. More efficient and cheaper protocols to obtain iOL are desirable for the future to broaden our knowledge in human oligodendrocyte biology.

The methods described above (summarised in Table 1) entail the use of an isolated population. Other methods have been developed to mimic the complexity of the environment in which OPC and oligodendrocytes reside, such as mixed glia cultures [144], organotypic brain slices [145], and iPSC derived organoids [146]. These models are useful to study the impact of other cell types on OPC and oligodendrocytes, to decipher any indirect effect exerted by other CNS cells and to complement in vivo studies.

### 4.2. In Vivo Models

One of the most used strategies to study oligodendroglial lineage cells is to genetically label markers typical of the lineage or of the maturation stage of interest with fluorescent proteins (see Table 1). These are particular suitable models to study the biology of oligodendrocytes in the context of a complex biological system. The most used species used for this purpose are mice or zebrafish. Mice generated to detect OPC during development and maturation are for example Sox10-Venus [147] or CNP-EGFP mice [148]. Other mice were generated targeting specific proteins, such as PLP-EGFP mice [149]. In recent years, zebrafish became popular as a model to study remyelination, due to some unique features: large numbers of offspring that develop quickly and outside of the mother, the transparency and small size of the animals that make live imaging of genetically modified zebrafish possible, the reduced costs and reduced level of self-consciousness compared to mammalian models. In fact, zebrafish share 70% of the genome with humans and most of the genes related to myelin are conserved [152,153]. Different transgenic zebrafish have been developed to study different stages and function of OPC and oligodendrocytes such as Tg(sox10:mRFP), Tg(olig2:EGFP) and Tg(mbp:EGFP) [103,150,151]. Given the consistency of the model, transgenic zebrafish are also suitable for high-throughput screening: recently, Early et al. developed an automatic analysis tool where zebrafish larvae are automatically delivered to a spinning disk confocal microscope and the images handled by an image analysis pipeline, facilitating the screening of pro-myelinating compounds [154].

### 4.3. Animal Models to study Oligodendrocytes and Remyelination in Multiple Sclerosis

There are no animal models that entirely recapitulate all of the features of MS, but different murine models are established to investigate different aspects of the pathology [155]. Experimental autoimmune encephalomyelitis (EAE) is an acute demyelinating episode triggered by the immune system stimulated by myelin protein with adjuvants [156]. EAE is a reproducible, well established model with a defined clinical pattern making it suitable to investigate the relevance of different molecular pathways for MS progression or the efficacy of new treatments.

Two models of non-immune-mediated demyelination use either dietary given cuprizone (a copper chelator) or injected lysolecithin (a lipidolitic detergent) [157,158]. Although the initiation of these models lacks immune engagement, immune cells are recruited to the demyelinated areas, indicating the importance of these cells also in the repair phase [159,160]. These models do not recapitulate any of the typical symptoms that patients with MS experience, but are very useful to investigate molecular and cellular mechanisms involved in the damage and repair phase. Cuprizone is a toxin that induces acute demyelination and is characterised by a peak of OPC infiltration 4 weeks after the start of the cuprizone diet in the corpus callosum, one of the most demyelinated areas in this model [161,162]. The infiltrated OPC are able to differentiate into oligodendrocytes in the following 2 weeks, representing the most abundant population after 6 weeks of cuprizone diet [162]. However, remyelination starts only after cuprizone is withdrawn: in one week, indeed, it is possible to observe an increased expression of myelin associated markers in the corpus callosum that stabilises upon complete remyelination 5 weeks after the re-establishment of a normal diet [162]. Interestingly, Sachs et al. recently developed a modified protocol able to slow down remyelination by the administration of rapamycin, an mTOR inhibitor, to the cuprizone treated mice, giving an extended temporal window to study the processes involved in myelin regeneration [162].

Lysolecithin induced demyelination is also a valuable model to study the remyelination process given the well described and distinct phases of OPC recruitment and proliferation in the lesion site between day five and 10 after the lesion induction, OPC differentiation between day 10 and 14 followed by myelin regeneration [83,158,163,164]. This well-established model is suitable for distinguishing the effect of molecules and cells of interest, including oligodendrocytes, in the different phases of myelin regeneration in an accurate manner.

The aforementioned models are able to recapitulate some features of MS; a comprehensive in vivo model reflecting the complexity of the disease is still lacking. Combination models have been developed to align animal models closer with human disease. For example, Rüther et al. combined cuprizone induced demyelination with EAE to induce a chronic model with persistent clinical signs and chronic lesions even in the forebrain, a region that is not usually affected in EAE, and with a robust immune component [165].

## 5. Oligodendrocytes in Disease

### 5.1. Oligodendrocytes in Diseases with Myelin Pathology

Oligodendrocyte death is not necessarily a sign of disease. Oligodendrocytes can die throughout development and adulthood without underlying pathology to enable neuronal plasticity and lifelong learning [166,167]. However, OPC and particularly oligodendrocytes are highly vulnerable to oxidative stress due to low antioxidant levels, for instance glutathione, and high iron content, which is required for enzyme activity. Iron in combination with hydrogen peroxide, from dismutation of superoxide, can produce highly reactive hydroxyl radicals via the Fenton reaction [168].

Especially during myelination oligodendrocytes are vulnerable to cytotoxic by-products from a high metabolic turnover (e.g., reactive oxygen species (ROS), hydrogen peroxide) [169,170]. Likewise, oligodendrocytes are susceptible to excitotoxicity from high glutamate and ATP concentrations [171,172]. Due to this vulnerability oligodendrocytes are affected in a range of disorders.

The most common pathological causes of oligodendrocyte death in the CNS are trauma, ischaemia or autoimmune attacks. Oligodendrocyte death can lead to subsequent demyelination or it can follow as a result of primary myelin damage [173]. In traumatic injuries and ischaemia, oligodendrocyte death and demyelination can follow the original injury [120,174], whereas in autoimmune diseases such as MS, oligodendrocytes are a primary target of an immune attack against myelin and oligodendrocyte specific proteins.

Much rarer are genetic defects that lead to oligodendrocyte damage as seen in some leukodystrophies [175]. The main cause of oligodendrocyte death in these diseases seem to be the accumulation of mutated PLP1, a prevalent protein in the myelin sheath, that fails to be transported to the plasma membrane leading to apoptosis of the cell [176].

White matter pathology is a characteristic of Alzheimer’s disease (AD), however oligodendrocyte death and demyelination are believed to occur secondary to neurodegeneration [177]. In post mortem AD tissue Olig2^+^ oligodendrocyte lineage cells were decreased [178] as were myelinating oligodendrocytes in a mouse model of AD [179]. The underlying cause is not established; however toxicity of beta-amyloid is likely a contributing factor [179,180]. Yet, oligodendrocyte pathology can be evident even before any neurodegenerative events materialise as a study by Fischer showed [181]. Intriguingly, WM pathology in AD is predominately affecting those CNS regions that were myelinated last during development (neuropathologic retrogenesis) [182,183], suggesting a connection between late myelin development and AD.

While oligodendrocyte pathology is not regarded as the primary cause, oligodendrocytes are downstream targets in some neuropsychiatric disorders including schizophrenia, bipolar disorders, autism, ADHD, mood disorders and depression (reviewed in [184] and [185]). For instance, oligodendrocyte density was region-specifically reduced in patients with bipolar disorders and schizophrenia [186,187,188], and animal models to study demyelination, such as the cuprizone model, have also been used to model aspects of schizophrenia and anxiety disorders (reviewed in [189]). In a chronic stress model oligodendrocyte specific genes were downregulated in the amygdala and prefrontal cortex [190], while dysregulation of oligodendrocytes and nodes of Ranvier is associated with depression [191].

### 5.2. Oligodendrocytes in Demyelination

Demyelinating diseases are the most common pathologies affecting oligodendrocytes. The most common demyelinating disease is MS [192]. MS is characterised by an immune-mediated attack against myelin sheaths and oligodendrocytes, primarily by myelin-specific CD8^+^ T cells [193]. This results in demyelination, which is characterised by destruction of the myelin sheath and death of oligodendrocytes [194] leaving axons denuded and vulnerable to neurodegeneration [1].

Genetic fate-mapping studies allowed adult OPC differentiation fates to be tracked after demyelination. This confirmed that adult OPC that represent approximately 6% of the total CNS cell numbers [48] are the main source of new oligodendrocytes after myelin damage [195]. In response to demyelination, adult OPC undergo a switch to an activated state that is characterised by an increased expression of numerous transcription factors [196,197]. These include SOX2 and TCF7L2, the latter of which is central to WNT signalling and maintains OPC in the cell cycle to enable proliferation and colonisation of areas of demyelination [2,197]. OPC migrate to the lesion, guided by microglia and astrocyte-derived factors including fibroblast growth factor and brain-derived neurotrophic factor [2,198,199], before differentiating into mature oligodendrocytes by exiting the cell cycle [200], a conversion led by the transcription factor E2F1 and proto-oncogene MYC [201,202]. The new oligodendrocyte is able to replace the destroyed myelin with a shorter and thinner myelin sheath [157] as shown in Figure 2.

### 5.3. Oligodendrocytes in Ageing

Ageing is associated with increased white matter atrophy, a decline in motor learning and diminished remyelination capacity [203,204]. The latter is due to changes in the ageing CNS environment [204] and to the decreased ability to recruit OPC that subsequently fail to differentiate into oligodendrocytes [205,206]. Rejuvenation can restore the reparative capacity in the CNS as the Franklin lab has shown: in a parabiosis experiment with young and old mice that were subjected to demyelination macrophages were key to restore the remyelination capacity in old animals by clearing myelin debris [204].

Due to their high metabolic rate during myelination, for the maintenance of myelin and for the trophic support of axons [117] oligodendrocytes are exposed to prolonged periods of cytotoxic by-products such as ROS; for instance, cholesterol synthesis is highly energy intensive [207] and also declines with age [208]. Additionally, compared to astrocytes oligodendrocytes have reduced antioxidants levels [169,209], which further decline with age [210]. Oligodendrocytes are highly vulnerable to oxidative stress, which can result in DNA damage [211]. With age, DNA repair mechanisms are either not efficient or are disturbed thus leading to oligodendrocyte apoptosis. As OPC recruitment and differentiation into oligodendrocytes is diminished with age [205,212], dead oligodendrocytes are not as efficiently replaced in old age as they were in younger age leading to white matter atrophy over time.

## 6. Oligodendrocytes in Myelin Regeneration

Remyelination is a natural regenerative process, which is believed to prevent neurodegeneration and restore function [2,213]. In MS and animal models of MS, the ability to remyelinate efficiently declines with age and disease progression (reviewed in [214]). Our knowledge about the mechanisms involved is mainly based on animal studies, which although powerful, deliver a simplified picture of what occurs in humans. This becomes increasingly clear with results from recent cell population studies in human healthy and MS brain tissue demonstrating the existence of different oligodendrocyte lineage cells, dependent on location, origin, disease phenotype and age [215,216,217]. New functions of oligodendrocyte lineage cells beyond their central roles in myelination and trophic support were recently identified including immunomodulatory properties as shown by Falcao et al. [126]. Given that MS is an immune-mediated disease, it would be striking if oligodendrocytes are not just the targets of an immune attack in MS but are actively involved in disease pathology.

OPC are central to remyelination by generating new oligodendrocytes that replace those lost after injury [218,219]. In contrast, surviving adult oligodendrocytes were long thought to not be involved in remyelination. This dogma has recently been revised for both rodents and humans as studies by Duncan et al. and Yeung et al. demonstrated [215,220]. Using rodent and non-human primate animal models, Duncan et al. showed that oligodendrocytes are connected to both myelinated and remyelinated myelin sheaths indicating that existing oligodendrocytes contribute to remyelination [220]. Comparing C14 in genomic DNA with atmospheric C14, Yeung et al. calculated the age of oligodendrocytes in MS lesions. The authors concluded that rather than newly formed oligodendrocytes from OPC old ‘spared’ oligodendrocytes regenerated myelin in remyelinated MS lesions [215].

Remyelination seems to replicate aspects of developmental myelination, at least in parts. For instance, similar transcription factors are involved in both processes, such as Myrf and Zfp488 [221,222]. Conversely, while Klf9 and Stat3 seem non-essential in developmental myelination they are critical for efficient remyelination [223,224] and Arnett et al. showed that the transcription factor Olig1 is more important than Olig2 in remyelination suggesting their role is reversed compared to developmental myelination [83].

In human MS lesions, Chang et al. and Kuhlmann et al. identified a block in OPC differentiation into oligodendrocytes as the bottleneck for efficient remyelination in MS [225,226]. In early MS and in successfully remyelinated lesions, differentiated oligodendrocytes are found within WM lesions, while in chronic stages of the disease, which is characterised by predominantly non-remyelinating lesions, differentiated oligodendrocyte are rare [225]. Albeit in lower numbers, OPC are still found in these chronic MS lesions indicating that OPC deficiency is not the primary cause for remyelination failure; it can, however, contribute to the impaired differentiation of OPC into remyelinating oligodendrocytes [225,227].

A study by Boyd et al. showed that deficiency of OPC within lesions either due to impaired recruitment and/or proliferation is another cause of remyelination failure for 37% of MS lesions, predominantly chronic active lesions. OPC deficiency in these lesions is linked to Semaphorin 3A that prevents OPC migration into the lesion area [205,228].

To overcome remyelination failure in MS new targets to enhance remyelination therapeutically are currently examined. Originally thought to only contribute to the pathology of MS, the immune system is now recognised in facilitating remyelination. Depletion of macrophages or lymphocytes leads to impaired remyelination following toxin-induced demyelination in animal models [159,229]. For instance, M2 macrophages and microglia promote oligodendrocyte differentiation via activin-A [230] and we showed that regulatory T cells promote oligodendrocyte differentiation and remyelination via CCN3 secretion [231].

## 7. Concluding Remarks

Oligodendrocytes have come a long way from the original description as ‘Nervenkitt’ to their central importance as the myelinating cells of the CNS. Adding to this key function, oligodendrocytes provide trophic support to axons and more recently, likely have additional immunomodulatory capacity. While we have a good understanding of the role of oligodendrocytes in myelination and remyelination, there is still a gap of knowledge of the molecular mechanisms involved in these processes. Given the similarities between myelination and remyelination, a better understanding of myelination can provide clues that can be harnessed to improve myelin regeneration, an unmet need for the treatment of demyelinating diseases, such as MS.

Only recently, new roles of oligodendrocytes have been uncovered such as potential immunomodulatory functions, which warrant further investigation, particularly in disease context. For instance, the question of whether oligodendrocytes are actively participating in disease progression e.g., in MS, by perpetuating inflammation would be interesting to address.

Advances in the models to study oligodendrocyte biology and function, such as cell population-based approaches, has broadened our knowledge beyond their classical roles.

Despite the mechanistic similarities between animals and humans in development and maturation of OPC and oligodendrocytes, there are differences that need to be accounted for when translating results to the human system. Different in vitro approaches based, e.g., on human iPSC will help to overcome the intrinsic interspecies differences that can complement and validate the discoveries from animal models.

In conclusion, oligodendrocytes have long left behind the ‘Nervenkitt’ attribute and are recognised as critical regulators of neuronal functions in CNS development, homeostasis and regeneration.

## Figures and Tables

**Table 1:** Summary of animal and human models listed in Section 4.1 and Section 4.2. This list provides examples of a variety of models available to study OPC and oligodendrocyte (OL) biology.

In vitro Animal Models	In vitro Human Models	In Vivo Animal Models
OPC/OL cultures [128,129,130]	OPC derived from biopsies [138] or post mortem CNS [139]	Reporter mice (e.g., Sox10-Venus mice, CNP-EGFP mice, PLP-EGFP mice) [147,148,149]
Immortalized oligodendroglial cells lines (e.g., CG-4, OLN-93, Oli-neu) [133,134,135]	iOL derived from human iPSC [137,141,142,143]	Reporter zebrafish (e.g., Tg(sox10:mRFP), Tg(olig2:EGFP), Tg(mbp:EGFP)) [103,150,151]

OPC: Oligodendrocyte Progenitor Cell, OL: oligodendrocyte, CNS: Central Nervous System, iOL: induced Oligodendrocyte, iPSC: induced Pluripotent Stem Cell.
